# Integration of Apo-α-Phycocyanin into Phycobilisomes and Its Association with FNR_L_ in the Absence of the Phycocyanin α-Subunit Lyase (CpcF) in *Synechocystis* sp. PCC 6803

**DOI:** 10.1371/journal.pone.0105952

**Published:** 2014-08-25

**Authors:** Pengpeng Zhang, Laurie K. Frankel, Terry M. Bricker

**Affiliations:** Department of Biological Sciences, Biochemistry and Molecular Biology Division, Louisiana State University, Baton Rouge, Louisiana, United States of America; Arizona State University, United States of America

## Abstract

Phycocyanin is an important component of the phycobilisome, which is the principal light-harvesting complex in cyanobacteria. The covalent attachment of the phycocyanobilin chromophore to phycocyanin is catalyzed by the enzyme phycocyanin lyase. The photosynthetic properties and phycobilisome assembly state were characterized in wild type and two mutants which lack holo-α-phycocyanin. Insertional inactivation of the phycocyanin α-subunit lyase (Δ*cpcF* mutant) prevents the ligation of phycocyanobilin to α-phycocyanin (CpcA), while disruption of the *cpcB/A/C2/C1* operon in the CK mutant prevents synthesis of both apo-α-phycocyanin (apo-CpcA) and apo-β-phycocyanin (apo-CpcB). Both mutants exhibited similar light saturation curves under white actinic light illumination conditions, indicating the phycobilisomes in the Δ*cpcF* mutant are not fully functional in excitation energy transfer. Under red actinic light illumination, wild type and both phycocyanin mutant strains exhibited similar light saturation characteristics. This indicates that all three strains contain functional allophycocyanin cores associated with their phycobilisomes. Analysis of the phycobilisome content of these strains indicated that, as expected, wild type exhibited normal phycobilisome assembly and the CK mutant assembled only the allophycocyanin core. However, the Δ*cpcF* mutant assembled phycobilisomes which, while much larger than the allophycocyanin core observed in the CK mutant, were significantly smaller than phycobilisomes observed in wild type. Interestingly, the phycobilisomes from the Δ*cpcF* mutant contained holo-CpcB and apo-CpcA. Additionally, we found that the large form of FNR (FNR_L_) accumulated to normal levels in wild type and the Δ*cpcF* mutant. In the CK mutant, however, significantly less FNR_L_ accumulated. FNR_L_ has been reported to associate with the phycocyanin rods in phycobilisomes via its N-terminal domain, which shares sequence homology with a phycocyanin linker polypeptide. We suggest that the assembly of apo-CpcA in the phycobilisomes of Δ*cpcF* can stabilize FNR_L_ and modulate its function. These phycobilisomes, however, inefficiently transfer excitation energy to Photosystem II.

## Introduction

The primary photoreactions of oxygenic photosynthesis are catalyzed by two major membrane protein complexes, Photosystem I (PS I) and Photosystem II (PS II). While these photosystems both have internal chlorophyll antennae, productive photosynthesis requires additional light-harvesting components. In cyanobacteria, as well as the eukaryotic classes Rhodophyta and Glaucophyta, these are the phycobilisomes. Phycobilisomes are large, highly structured peripheral water-soluble complexes consisting of an allophycocyanin core which is attached to multiple oriented rods. These rod elements are composed of phycocyanin, phycoerythrin and phycoerythrocyanin (the exact composition being species-dependent) and their associated linker polypeptides. Recently, it has been demonstrated that phycobilisomes can physically associate with, and transfer excitation energy to, both Photosystem II and I [1], [2]. Covalent attachment of the phycobilin chromophores to specific cysteinyl residues of the apo-phycobiliproteins via a thioether bond is catalyzed by phycobilin lyases [3], [4]. *Synechocystis* sp. PCC 6803, henceforth *Synechocystis*, has a relatively simple phycobilisome structure containing only the allophycocyanin core and phycocyanin-containing rods. The heterodimeric CpcE/CpcF lyase is specifically responsible for phycocyanobilin attachment to the α subunit of phycocyanin (CpcA) [5], [6].

In both the cyanobacteria and chloroplasts, the role of ferredoxin: NADP^+^ oxidoreductase (FNR) is to catalyze the final step of photosynthetic electron transport, providing reducing equivalents in the form of NADPH for CO_2_ fixation and other reductive metabolic pathways. Higher plants contain multiple FNR genes encoding a variety of different isoforms of this enzyme. In cyanobacteria, however, FNR is encoded by a single gene, *petH*. The FNR in most phycobilisome-containing cyanobacteria has an N-terminal conserved domain which shares high sequence similarity with the phycocyanin rod linker polypeptide CpcD. This domain appears to be responsible for attachment of FNR to the peripheral rods in phycobilisomes [7], [8]. Recent studies indicate that in cyanobacteria two FNR isoforms (FNR_L_ and FNR_S_) are produced from the single *petH* gene via alternative transcriptional start points and, consequently, different translation initiation sites [9], [10]. Under normal photosynthetic conditions, FNR_L_ is present as the major isoform, while FNR_S_ is induced under a variety of stress conditions such as iron starvation [9]. An FNR_L_-phycocyanin complex has been purified from *Thermosynechococcus elongatus* and *Synechocystis* and the enzymatic activities have been characterized *in vitro* [11], [12].

In this work, we characterized two phycocyanin defective strains of *Synechocystis*, a phycocyanin α-subunit lyase mutant (Δ*cpcF*) and a *cpcB/A/C2/C1* operon deletion mutant (CK) to address how the assembly of phycocyanin into the phycobilisome regulates photosynthetic performance via light energy absorption and downstream energy utilization. Interestingly, we find that apo-CpcA appears to assemble into phycobilisomes containing holo-CpcB, linker polypeptides and the allophycocyanin core. While these mutant phycobilisomes inefficiently transfer excitation energy to the photosystems, they can associate with FNR_L_ and stabilize this component.

## Material and Methods

### Strains and cell culture conditions

A glucose-tolerant strain of *Synechocystis* [13] was used as the wild-type strain for this study. The phycocyanin α-subunit lyase inactivation mutant Δ*cpcF* was constructed by insertion of a kanamycin-resistant cassette at the position 193 of *sll1051*. After selection, segregation of the mutant allele was verified by PCR (data not shown). The insertion mutation was tested for polar effects both down- and up-stream of the *sll0151* gene; none were identified (data not shown). The WT, Δ*cpcF*, and phycocyanin-null mutant, CK [9], were generally grown in BG-11 medium buffered with 10 mM TES-KOH (pH 8.2), except for low CO_2_ growth conditions where Na_2_CO_3_ was omitted from the BG-11 recipe and the medium was buffered with 20 mM HEPES-NaOH (pH 7.5). The mutant strains were maintained in the presence of 10 µg/ml kanamycin. The strains were grown either photoautotrophically, photomixotrophically, or photoheterotrophically. For photomixotrophic growth, 5 mM glucose was added to the basal BG-11 medium. For photoheterotrophic growth, BG-11 medium was supplemented with both 5 mM glucose and 10 µM 3-(3,4-dichlorophenyl)-1,1-dimethylurea (DCMU). The cells were grown under continuous white fluorescent illumination with light intensity of 50 µmol photons m^−2^s^−1^ at 30°C. The strains were grown either on 1.5% (w/v) agar plates, or in liquid culture aerated by bubbling with sterile and humidified air. To achieve a CO_2_ concentration lower than air levels, the air flux was passed through Ascarite II (Sigma-Aldrich) at a constant rate and the CO_2_ concentration was monitored by infrared gas analysis.

### Oxygen evolution measurements

Light response curves were measured by recording steady state oxygen evolution rates of the cells under illumination with different light intensities using a Clark-type oxygen electrode (DW1, Hansatech) at 26°C. The WT, Δ*cpcF* and CK strains were grown autotrophically at ambient CO_2_, were collected by centrifugation, washed, and resuspended in fresh growth medium at a chlorophyll concentration of 10 µg/ml. Chlorophyll concentration was determined as described by Williams [13]. The measurements were performed with either actinic white light or 650 nm red light. Net photosynthesis was measured in the presence of 10 mM NaHCO_3_ as a terminal electron acceptor. The PS II electron transfer rates from H_2_O to quinone were measured in the presence of 0.5 mM 2,6 dichloro-*p*-quinone (DCBQ) +1 mM K_3_Fe(CN)_6_.

### Fluorescence measurements

Chlorophyll fluorescence induction was monitored with a dual-modulated fluorometer FL 3000 (PSI Instruments). The cells were diluted to a chlorophyll concentration of 5 µg/ml. Measurements were taken after 5 min dark adaptation, followed by illumination with the highest level of actinic light (1000 µmol photons m^−2^s^−1^, 625 nm). The oxidation state of the plastoquinone pool was modulated by the addition of either 15 µM DCMU or 20 µM 2,5-dibromo-3-methyl-6-isopropylbenzoquinone (DBMIB) in the fluorometer cell after dark adaptation. Data were collected with measuring light in a logarithmic series between 10 µs to 1 s. The fluorescence curves were plotted directly or normalized according to the equation Fluorescence  =  (1-F_0_/Ft)/(F_V_/F_M_) [14]. Data were analyzed using Origin version 8.1 and proprietary software provided by Photon Systems Instruments.

### Isolation of membrane and soluble fractions

*Synechocystis* membrane and soluble fractions were isolated according to [15]. The cell cultures (150ml OD_730_ = 1) were pelleted at 4°C by 5 min centrifugation at 5,000×g. The cell pellet was washed twice with wash buffer containing 50 mM MES-NaOH pH 6.5, 50 mM NaCl, 10 mM MgCl_2_, 5 mM CaCl_2_, and resuspended in 1.5 ml wash buffer containing 25% (v/v) glycerol (break buffer). An equal volume of glass beads (Sigma, 150-212 µm diameter) was added to the cell suspension, which was chilled on ice. The cells were broken by manually vortexing eight times at maximal speed for 1 min at 4°C with 1 min cooling on ice between the cycles. The glass beads and cell debris were removed by centrifugation at 2,000×g for 5 min, and the supernatant was collected. The glass beads were washed with 1 ml break buffer and centrifuged for 5 min. The supernatant was combined with the previously collected samples. The membranes and soluble fractions were separated by centrifugation at 30,000×g for 30 min.

### Isolation of phycobilisomes

Phycobilisomes were isolated according to [16]. The cells were harvested from 0.5–1 L culture at OD_730_>2 and washed twice with 0.8 M potassium phosphate buffer, pH 7.0 (KP). The cell pellets (1 g in fresh weight) were resuspended in 3 ml KP in the presence of 1 mM 4-(2-aminoethyl)benzenesulfonyl fluoride hydrochloride (AEBSF). The cells were broken as described above by vortexing with glass beads. The broken cell extracts were incubated with Triton X-100 at a final concentration of 2% (v/v) for 20 min at room temperature in darkness with occasional gentle shaking. The unbroken cells and membrane debris were removed by centrifugation at 30,000×g for 20 min at 15°C. The supernatant was loaded onto a 10–35% (w/v) linear sucrose gradient in 0.8 M KP with 1 mM AEBSF, and centrifuged at 130,000×g for 24 h at 15°C. After centrifugation, the gradients were either fractionated into 30, 400 µl fractions, or the blue bands were collected directly with a syringe. The samples from the sucrose gradient were precipitated by the addition of an equal volume of 20% trichloroacetic acid and incubated on ice for 10 min. After centrifugation at 14,100×g for 10 min at 4°C, the pellets were washed twice with 100% acetone at 4°C to remove residual potassium phosphate. The pellets were then resuspended in LiDS gel-loading buffer for protein analysis.

### Electrophoresis and immunoblotting

Gradient (12.5–18%) LiDS-PAGE was used to analyze denatured protein samples according to Delepelaire and Chua [17]. Soluble protein complexes were analyzed by BN-PAGE as described in Zhang et al. [18] on a 6–13.5% gradient polyacrylamide gel. Samples were prepared at 4°C under low light illumination. No solubilization procedure was required for the soluble fraction samples. The protein samples were diluted with one third volume of a buffer containing 100 mM Bis-Tris, pH 7.0, 80% (w/v) glycerol, 0.08% *n*-dodecyl β-D-maltoside (DM), and 4 mM AEBSF prior to electrophoresis. After electrophoresis, the proteins or protein complexes were electroblotted onto a polyvinylidene fluoride membrane (PVDF) and labeled with protein- specific antibodies followed by chemilumenescent detection. Protein expression was semi-quantified by comparison with a dilution series of WT samples (5–75 µg protein). Signal intensities were analyzed by ImageJ [19]. These are shown in Fig. S1.

## Results

### Growth of wild type and phycocyanin mutant stains with different carbon sources

**Figure 1:**
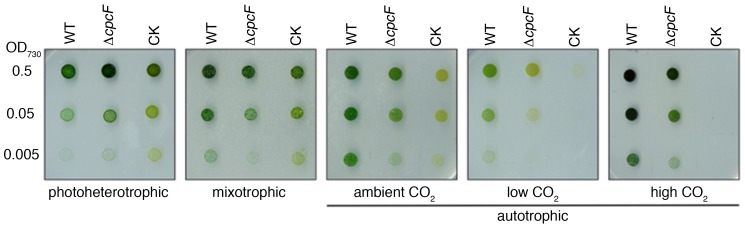
Growth of WT, Δ*cpcF* and CK strains on BG-11 agar plates with different carbon sources. Cells were grown autotrophically on air in BG-11 liquid cultures. They were then harvested by centrifugation and resuspended in fresh BG-11 medium. The concentration of the cells was adjusted to OD_730_ = 0.5, 0.05 and 0.005. For each sample, 4 µl of the cell suspension were placed on the agar plate and the cultures were grown for 5–7 days. photoheterotrophic: BG-11 medium +5 mM glucose +10 µM DCMU; mixotrophic: BG-11 medium +5 mM glucose; autotrophic: BG-11 medium + air at different CO_2_ concentrations: ambient CO_2_, ≈500 ppm; low CO_2_, ≈220 ppm; high CO_2_, ≈5000 ppm.

To examine the cell viability and growth rate, wild type (WT) and the two phycocyanin-defective mutants, Δ*cpcF* and CK, were grown on BG-11 plates in the presence or absence of glucose and DCMU. All three strains had similar growth rates in the presence of glucose under either photoheterotrophic or mixotrophic conditions. Under autotrophic growth conditions (≈500 ppm CO_2_), however, both the Δ*cpcF* and CK mutants grew slower than WT (Fig. 1). The slower growth rates of the mutants suggested a defect in photosynthesis. These strains were then examined during autotrophic growth under both low (≈220 ppm) and elevated (≈5000 ppm) CO_2_ concentrations. Both the WT and Δ*cpcF* strains exhibited enhanced growth at a high CO_2_ concentration. Interestingly, the CK mutant grew very poorly under both high and low levels of CO_2_ (Fig. 1).

### Photosynthetic light response curves of WT and phycocyanin mutants

**Figure 2:**
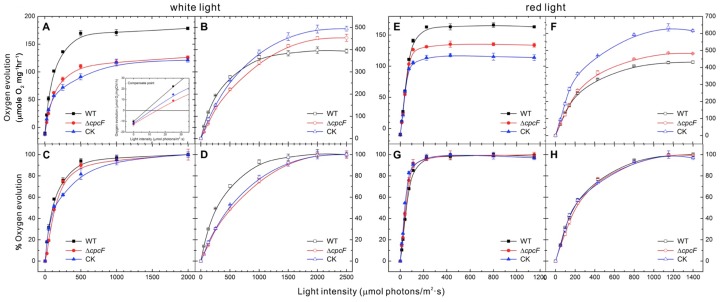
Light saturation curves of oxygen evolution for WT, Δ*cpcF* and CK strains under white and red actinic light illumination. Oxygen evolution rates were measured with cell suspensions at a chlorophyll concentration of 10 µg/ml in BG-11 medium using different intensities of either white light (A–D) or red light (E–G). Net photosynthesis was measured in panels A, C, E and G (H_2_O to CO_2_). PS II activity was measured in B, D, F and H (H_2_O to DCBQ). The upper panels A, B, E and F show the absolute oxygen evolution rates observed. In Panels C, D, G and H the data were normalized to the maximum rates observed in Panels A, B, C and D, respectively. The error bars represent ±1.0 standard deviation (SD) and in some instances were smaller than the symbols; each experiment is the average of 3–4 independent measurements.

To examine the overall photosynthetic property of these strains, oxygen evolution measurements were determined at different intensities under both white and red light (650 nm) illumination conditions. Under white actinic light illumination, the maximal oxygen evolution capacity from water to CO_2_ of both phycocyanin mutants is lower than observed in WT (Fig. 2A), suggesting that the overall photosynthetic capacity of the Δ*cpcF* and CK mutants is diminished in these strains. The light compensation point (Fig. 2A, insert) and examination of the light saturation curves (Fig. 2A and C) indicated that the mutant strains saturate photosynthesis at somewhat higher light intensities than does WT. These results indicate that the white light absorption efficiency is lower in mutant cells than in WT. PS II oxygen evolution rates using DCBQ as an artificial electron acceptor were also examined (Fig. 2B and D). Both mutant strains exhibited higher maximal rates of oxygen evolution than WT. This was not unexpected, as mutants depleted in phycobilisomes accumulate increased amounts of PS II [20]. The normalized PS II light saturation curves (Fig. 2D) indicate that the Δ*cpcF* and CK mutants are nearly identical (Fig. 2D) suggesting that the mutants have similar white light-trapping efficiencies. The light intensity providing ½ maximal PS II activity was 500 µmole photons⋅m^−2^⋅sec^−1^ for both mutants and 300 µmole photons⋅m^−2^⋅sec^−1^ for WT. This indicates that WT is significantly more efficient at energy capture than the Δ*cpcF* and CK mutants when white light is used as an illumination source. These results suggest that, as expected, both phycocyanin mutants have defective light absorption properties when compared to the WT strain. These defects probably arise from the lack of functional phycocyanin-containing rod elements in both mutants.

Additionally, we measured photosynthetic activity using a red LED actinic light source (650 nm). The allophycocyanin core of the phycobilisome preferentially absorbs at this wavelength. The maximal rate of oxygen evolution, using CO_2_ as a terminal electron acceptor under this illumination condition, was higher in WT than in either of the phycocyanin mutants (Fig. 2E), similar to what was observed in white light (Fig. 2A). The normalized light saturation curves of the mutants, however, were very similar to that of WT (Fig. 2G). Examination of PS II activity using DCBQ as an electron acceptor again demonstrated that the mutants exhibited higher rates of electron transport than WT, as was observed under white light illumination (Fig. 2B). The normalized light saturation curves indicated that WT and the phycocyanin mutants were equally efficient at harvesting 650 nm red light. These results indicate that WT and both mutants appear to have functionally equivalent light absorption properties in red light, suggesting that all three strains have fully functional allophycocyanin cores associated with their phycobilisomes.

**Figure 3:**
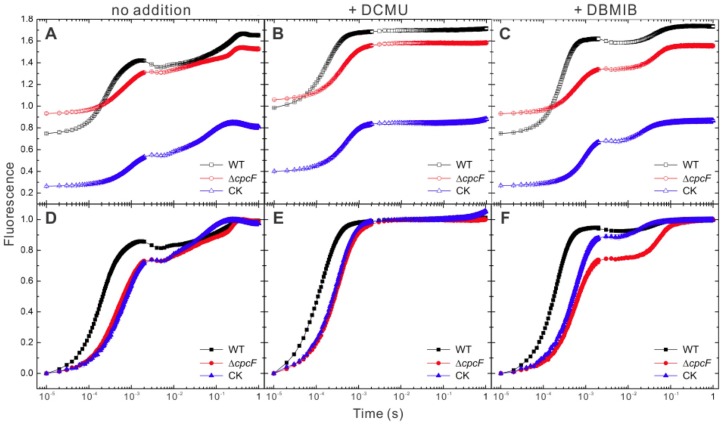
Chlorophyll fluorescence induction of WT, Δ*cpcF* and CK strains. The cells were grown autotrophically at ambient CO_2_. Chlorophyll fluorescence induction of dark-adapted cells at a chlorophyll concentration of 5 µg/ml in BG-11 medium was measured using 1000 µmol photons/m^2^·s orange-red light (625 nm). The chlorophyll fluorescence was presented either as the raw fluorescence traces (A–C) or normalized using the equation Fluorescence  =  (1-F_0_/F(t))/(F_V_/F_M_) (D–F). The samples for the measurements contained either no additions (A and D), or were performed in the presence of 15 µM DCMU (B and E) or 20 µM DBMIB (C and F). The error bars represent ±1.0 SD and in some instances were smaller than the symbols; each experiment is the average of 3 independent measurements.

We also examined the chlorophyll fluorescence kinetics of WT and the phycocyanin deficient strains over the first 1.0 s of actinic illumination (Fig. 3, Table 1, Fig. S2). WT exhibited an OJIP fluorescence transient curve typical of those observed previously in WT strains [21], [22]. While the variable fluorescence (F_V_) of the Δ*cpcF* and CK mutants were similar, and smaller than that of WT, their initial fluorescence levels (F_0_) were quite different. Δ*cpcF* exhibited a higher F_0_, while the CK strain had a much lower F_0_ value than that observed for WT (Fig. 3A, Table 1). To examine the effect of the redox state of the plastoquinone pool on the OJIP transient, DCMU (Fig. 3B) and DBMIB (Fig. 3C) were supplied to the dark-adapted cell suspensions prior to collection of the fluorescence transient data. In the presence of DBMIB there was no significant change observed in the F_0_ levels for any of the strains examined. In the presence of DCMU all of the strains exhibited higher F_0_ values. Both DCMU and DBMIB slightly increased F_M_ levels of WT and Δ*cpcF* cells, while the F_M_ of CK appeared insensitive to these treatments (Fig. 3A, B and C, Table 1). Normalization of these fluorescence transients to (F =  (1-F_0_/F(t))/(F_V_/F_M_)) [14] indicated that the OJ transition occurred slower in the Δ*cpcF* (T_J_ = 2.63 ms) and CK (T_J_ = 3.19 ms) mutants than in WT (T_J_ = 1.67 ms) and were of lower magnitude (Fig. 3D, Table 1). The differences in the OJ transients were not eliminated in the presence of DCMU (Fig. 3E, Table 1) or DBMIB (Fig. 3F, Table 1) suggesting that the phenomenon arose from the donor side of PS II. In all cases the T_J_ for the mutants were significantly slower than that observed for WT. Decreasing the actinic light intensity for WT samples nearly eliminated the observed alterations in both the magnitude of the OJ transition and its kinetics (Fig. S2). Consequently, we hypothesize that the observed differences in the OJ transitions are due to the mutants having a smaller optical cross-section brought about by alterations in the phycobilisome antennae. Treatment with DBMIB increased the J peak amplitude of WT and the CK mutant, while the J peak amplitude was essentially unchanged in the Δ*cpcF* strain. (Fig. 3F). Interestingly, the T_P_ values for WT and the Δ*cpcF* mutant were nearly identical in both the absence and presence of DBMIB. The T_P_ value for the CK mutant was lower under both control conditions and in the presence of DBMIB treatment (Table 1). These results indicated that the plastoquinone pool became reduced somewhat more rapidly in the CK mutant than in WT and the Δ*cpcF* strain. This may result from defective NADP^+^ reduction in the CK strain (see below).

**Table 1:** Chlorophyll fluorescence parameters during the OJIP transition.

Strain	F_0_	F_M_	F_V_	F_V_/F_M_	T_J_ (ms)	T_P_ (s)
No addition						
WT	0.75±0.01	1.67±0.01	0.92±0.01	0.55±0.01	1.67±0.01	0.41±0.01
Δ*cpcF*	0.93±0.01	1.54±0.01	0.61±0.01	0.39±0.01	2.63±0.04	0.39±0.01
CK	0.26±0.01	0.85±0.01	0.59±0.01	0.69±0.01	3.19±0.02	0.24±0.01
+ DCMU						
WT	0.99±0.01	1.70±0.01	0.72±0.01	0.42±0.01	4.00±0.05	NA
Δ*cpcF*	1.06±0.01	1.58±0.01	0.52±0.01	0.33±0.01	6.33±0.30	NA
CK	0.40±0.01	0.85±0.01	0.44±0.01	0.52±0.01	4.66±0.18	NA
+ DBMIB						
WT	0.75±0.01	1.74±0.01	0.99±0.01	0.57±0.01	1.83±0.01	0.37±0.01
Δ*cpcF*	0.93±0.03	1.56±0.01	0.63±0.01	0.40±0.01	2.73±0.04	0.36±0.01
CK	0.27±0.01	0.87±0.01	0.60±0.01	0.69±0.01	3.22±0.02	0.26±0.01

The WT and mutant strains were examined either with no addition or with the addition of 10 µM DCMU or 20 µM DBMIB. The values shown represent the mean ±1.0 SD; each experiment is the average of 3 independent measurements. NA, Not Applicable.

### Expression and assembly of phycobiliproteins and ferredoxin-NADP^+^ reductase in WT and the phycocyanin mutants

To examine the possibility that proper incorporation of phycocyanobilins into the phycocyanin protein may affect not only its function but also the stability, assembly and accumulation of other phycobilisome proteins, we examined the expression of a number of phycobiloproteins in WT and the phycocyanin mutant strains. These included CpcA, CpcB, and ApcB. CpcA and CpcB are major pigment-binding components of the phycobilisome rods, and ApcB is a component of the allophycocyanin core of the phycobilisome. CpcA is the substrate for the heterodimeric CpcE/CpcF phycocyanin α subunit lyase. We also examined the expression of FNR in these strains since cyanobacterial FNR_L_ has shown high affinity to the phycobilisomes, and its association with phycocyanin modulates FNR enzymatic activity [12].

**Figure 4:**
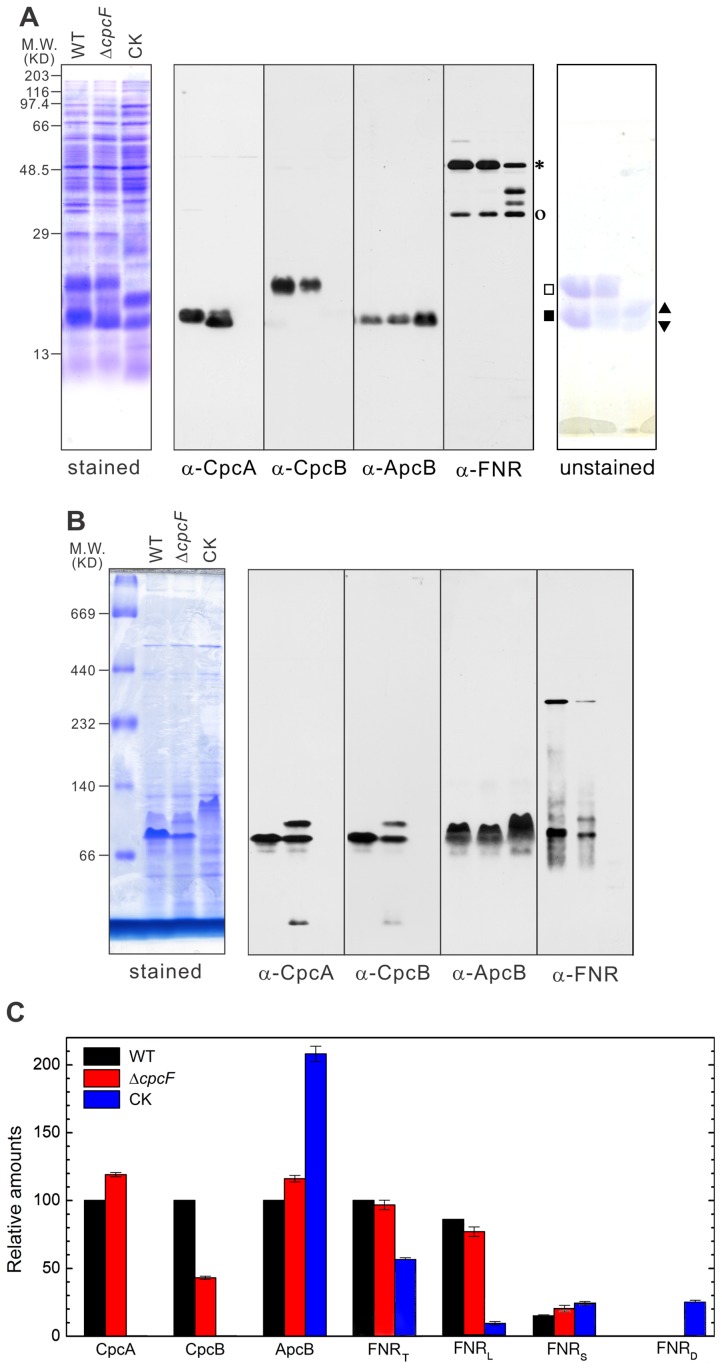
Expression and assembly of FNR and phycobiliproteins in WT, Δ*cpcF* and CK strains. Soluble proteins were separated on either (A) denaturing LiDS-PAGE or (B) BN-PAGE. Proteins were visualized either by Coomassie stain or immunodetection. (C) The grey scale levels of the bands obtained from the LiDS-PAGE immunoblots were semi-quantified and plotted. FNR_T_, FNR_L_, FNR_S_ and FNR_D_ are the amounts of total FNR, the large form of FNR, the small form of FNR and the degraded FNR observed, respectively. The standard curves used in this semi-quantification are shown in Fig. S1. The error bars represent ±1.0 SD; each experiment is the average of 3 independent measurements. Symbols: *, FNR_L_; ^o^, FNR_S_; ^▪^, CpcA; ^□^, CpcB; ^▴^, ApcA; ^▾^, ApcB.

The protein expression of the phycocyanin and allophycocyanin proteins as well as FNR are shown in Fig. 4. In the Δ*cpcF* mutant, the total amount of immunodetectable CpcA is slightly larger than that found in WT (Fig. 4A and C). However, the vast majority of this protein is found in a lower apparent molecular mass band, with only a small fraction migrating at the position of CpcA in WT. This appears to arise from the accumulation of apo-CpcA since, in the absence of the phycocyanin α-subunit lyase in the Δ*cpcF* mutant, the phycocyanobilin chromophores would be inefficiently attached to the protein. This is apparent in the unstained panel, as no major blue band is observed at this location in the Δ*cpcF* mutant. The amount of CpcB observed in the Δ*cpcF* mutant is significantly lower than that observed in WT (Fig. 4A and C) and appears to be fully pigmented. As expected, neither CpcA nor CpcB were present in the CK mutant, which lacks the genes encoding these two components. The Δ*cpcF* mutant accumulated slightly more ApcB than WT which is consistent with the presence of the allophycocyanin core of the phycobilisome in both of these strains; the CK mutant, however, exhibited substantially more ApcB than either WT or the Δ*cpcF* mutant (Fig. 4A and C). Analysis of the two forms of FNR in these strains indicated that the amount of FNR_L_ was significantly decreased in the CK mutant. Additionally, lower mass bands were observed which may represent degradation products of this component. Similar results have been reported previously for this strain [9]. The amount of FNR_S_ was slightly elevated in both phycocyanin mutant strains (Fig. 4A and C). These results suggest that the FNR_L_ is unstable in the CK mutant and that the presence of apo-CpcA in the Δ*cpcF* mutant appears to stabilize FNR_L_.

In order to investigate the interaction of FNR_L_ and CpcA and CpcB proteins, we used Blue-Native –PAGE (BN-PAGE) followed by immunodetection with FNR and Cpc antibodies (Fig. 4B). Due to the low phosphate concentration in the isolation buffer, the components of the phycobilisome partially dissociate. Under this condition, a putative phycobilisome subcomplex of about 80 kDa, which contained the CpcA, CpcB and FNR proteins, was detected in both WT and the Δ*cpcF* mutant. Additionally, a higher molecular mass band (≈300 kDa), which reacted with the FNR antibody, was also observed in these strains. In the CK strain a subcomplex which appeared to contain ApcB was observed at an apparent molecular mass of about 90 kDa. No bands were found to react with FNR antibodies in this strain. This appears to indicate that phycocyanin was required for the formation and/or stability of these FNR complexes (Fig. 4B). Importantly, these results imply that in the Δ*cpcF* mutant, apo-CpcA can assemble with FNR and CpcB to form a putative phycobilisome subcomplex and that this association appears to stabilize the FNR.

**Figure 5:**
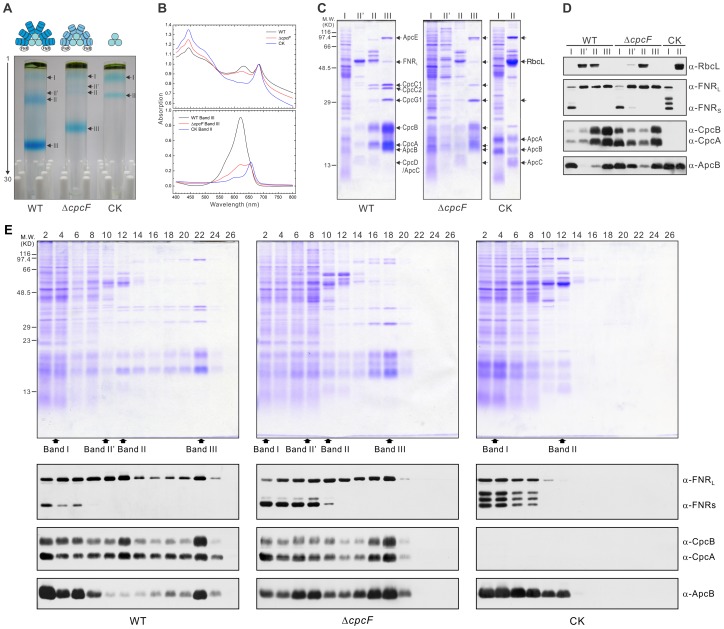
Phycobilisome profile analysis of the WT, Δ*cpcF* and CK. Phycobilisomes were isolated on a 10–35% sucrose density gradient. (A) The sucrose density gradients showing the resolved blue phycobilisome bands. (B) The absorption spectra of whole cells (upper panel) and the lowest phycobilisome band from the sucrose density gradients from each strain (lower panel) were measured at room temperature. The protein components of each band were separated by LiDS-PAGE and visualized by, C, Coomassie blue stain or, D, immunodetection. (E) The sucrose gradients were fractionated into 30 samples and subjected to LiDS-PAGE. Distribution of proteins within the sucrose gradients are shown in the upper panel by Coomassie blue stain and, in the lower panels, the distribution of FNR, CpcA, CpcB and ApcB are shown by immunodetection.

To examine the assembly state of the phycobilisomes in the phycocyanin mutants, intact phycobilisomes were resolved by sucrose density gradient centrifugation in the presence of a high phosphate concentration (Fig. 5A). Several blue bands were observed in the sucrose gradients (Bands I, II′, II, and III). Because of its position near the top of the gradient, Band I from all samples was contaminated with multiple proteins which did not penetrate the sucrose gradient (Fig. 5C). Since in all likelihood, Band I contained unassembled phycocyanin and allophycocyanin proteins, we focused on the compositions of Band II and Band III. The absorption spectrum of cells (Fig. 5B, top) and the lowest band from each sample, Band III for WT and the Δ*cpcF* mutant and Band II for the CK strain, are shown (Fig. 5B, bottom). These represent the largest phycobilisome assemblies present in these strains. Compared to WT, the phycocyanin absorption peak of the phycobilisome was significantly decreased in the Δ*cpcF* mutant strain and absent in the CK mutant. It should be noted that the phycocyanin absorption peak which we observed in the Δ*cpcF* mutant was much larger than that reported for the *Synechococcus* sp. PCC 7002 Δ*cpcE/F* mutants [6]. The phycobilisome which assembles in the Δ*cpcF* strain was smaller in size than that of WT (Fig. 5A). Lithium dodecyl sulfate-PAGE (LiDS-PAGE) analysis revealed that the phycobilisome in Δ*cpcF* had relatively lower amounts of CpcA, CpcB and the linker polypeptide CpcC1, an apparent absence of CpcC2, and more abundant CpcG1 (Fig. 5C). Interestingly, CpcC2 was also absent from phycobilisomes isolated from a *Synechocystis* phycocyanin β-subunit lyase (*cpc*T) mutant [23]. The absence of CpcC2 would suggest that no core-distal phycocyanin hexamers were present in the assembled phycobilisomes from the Δ*cpcF* strain. Due to lack of the phycocyanin proteins, Band III was absent in the CK strain and Band II contained the allophycocyanin core. This band migrated to a position which overlapped with Rubisco, which was also enriched in the WT Band II′ (Fig. 5D). It should be noted that dissociated allophycocyanin core did not accumulate in the WT strain; Band II in WT was larger than that observed in the CK mutant and appeared to consist mainly of partially assembled phycocyanin rod elements. The Band II in the Δ*cpcF* mutant was also smaller than that observed in WT. It contained phycocyanin proteins and a significant amount of ApcB was also detected. Consequently, Band II in Δ*cpcF* appears to be a mixture of phycocyanin rods and allophycocyanin core components (Figs. 5C–E).

In addition to the phycobilisome proteins, Bands II and III from both WT and Δ*cpcF* contained FNR_L_ (Figs 5C and D). Band II in the CK mutant did not exhibit any FNR protein (Fig. 5D). FNR_S_ seemed to be located at the top of the sucrose density gradient in all of the examined strains and did not appear to be associated with assembled phycobilisomes (Fig. 5D and E). These results indicate that the phycobilisomes present in the Δ*cpcF* mutant can associate with, and stabilize, FNR_L_. Additionally, these phycobilisomes appear to contain apo-CpcA. Apparently, integration of apo-CpcA can support the assembly of modified phycobilisomes in this strain.

## Discussion

### Proper assembly of phycocyanobilin pigments into the α-subunit of phycocyanin is required for optimal growth and light-harvesting

In cyanobacteria, phycocyanin is a major component of the phycobilisome light- harvesting apparatus. Holo-phycocyanin has a characteristic blue color, which is due to the covalent attachment of phycocyanobilin chromophores. Attachment of phycocyanobilins to the CpcA and CpcB apoproteins is catalyzed by a specific phycocyanin α-subunit lyase (CpcE/CpcF) and phycocyanin β-subunit lyases (CpcT and CpcS/CpcU), respectively [6], [24], [25]. It should be noted that while the attachment of phycocyanobilin can occur spontaneously, this is a very inefficient process [26]. In our study, inactivation of the *Synechocystis* 6803 *cpcF* gene in the Δ*cpcF* mutant and the absence of CpcA and CpcB in the CK mutant resulted in major growth (Fig. 1) and light-harvesting defects (Figs. 2–3). When examined under photoheterotrophic and mixotrophic growth conditions, the WT and mutant strains grew nearly equivalently (Fig. 1). Growth under autotrophic conditions, however, was compromised in the phycocyanin mutant strains. Under ambient and low CO_2_ conditions, both mutants grew poorly. This had been observed previously for other phycobilisome mutants including the CK mutant and a mutant completely lacking phycobilisomes [27]. It is possible that the absence of an intact phycobilisome affects the function of the Carbon Concentrating mechanism in *Synechocystis*. Interestingly, under the high CO_2_ growth condition, while the Δ*cpcF* mutant exhibited significant growth, the CK mutant exhibited no detectable growth on plates. Very slow growth had previously been observed for this strain in liquid culture [27] at high CO_2_ growth conditions. At this point in time we cannot provide a definitive explanation for this observation. It is possible, however, that the absence of phycobilisome-associated FNR_L_ in this strain (see below), severely compromises linear electron transport to NADPH. Under limiting NADPH conditions and at high CO_2_ concentrations, Rubisco would be operating at a maximal rate but the regeneration of ribulose 1,5-bis-phosphate could be problematic.

Functionally, the light saturation curves for net photosynthesis (H_2_O to CO_2_) and PS II activity (H_2_O to DCBQ) did not show significant differences between WT and two mutant strains when using 650 nm red actinic illumination (Fig. 2G and H). This indicates that the allophycocyanin phycobilisome core is functional in providing excitation energy to PS II in WT and both phycocyanin-deficient strains. However, when using white actinic light for these measurements, both mutants required a higher saturating light intensity, particularly for PS II activity (Fig. 2D). This suggested that the higher light requirement was a result of defective energy transfer from phycocyanin. Since the PS II light saturation curves of the lyase mutant Δ*cpcF* and phycocyanin-null mutant CK were similar (Fig. 2D), it appears that the phycocyanin which accumulates in the Δ*cpcF* strain is not functional in energy transfer despite the presence of holo-CpcB (Fig. 4A). This assertion is supported by the fluorescence induction experiments illustrated in Fig. 3. Both of the phycocyanin mutants exhibited a slow OJ transition when compared to WT under all conditions tested (Table 1, Fig. 3D–F). This appears to be due to defective energy transfer since, when WT was examined at a lower light intensity, this difference was nearly eliminated (Fig. S2). The lack of functional phycocyanin in both mutants appears to result in inefficient light-harvesting. Consequently, both mutants had lower maximal net photosynthesis rates than WT (Figs. 2A and E), which is in agreement with a slower growth rate under autotrophic conditions (Fig. 1). Interestingly, while the amount of CpcB protein in the Δ*cpcF* mutant dropped to less than half of the WT, the amount of CpcA showed a slight increase, even though no bilin chromophores were attached and this protein was present as apo-CpcA (Fig. 4A and D). The apo-CpcA protein accumulated and did not appear to be subject to rapid degradation, since it appeared to be assembled into phycobilisomes in the Δ*cpcF* mutant (Fig. 5A, D, E).

### Truncated phycocyanin rods in the Δ*cpcF* mutant stabilizes FNR_L_

The phycobilisomes of the Δ*cpcF* strain appeared to be similar to those found in WT, although smaller in size. They contained the allophycocyanin core subunits, CpcA and CpcB, and several linker peptides (Fig. 5A and C, Band III). While lacking CpcC2, the Δ*cpcF* phycobilisomes were enriched in CpcG1 (Fig. 5A and C, Band III). The lack of the CpcC2 linker may explain the smaller size of these phycobilisomes since CpcC2 appears to be required for the assembly of distal phycocyanin rod hexamers [28]. CpcC2 was also absent in a *cpcT* mutant which lacked the CpcB phycocyanin lyase [23]. Most interestingly, the phycobilisomes from the Δ*cpcF* mutant contained large amounts of CpcA (Fig. 5C and D). Since most of the CpcA present was in the unpigmented apo-CpcA form (Fig. 4A) it appears that significant quantities of apo-CpcA can assemble into phycobilisomes in the Δ*cpcF* the strain. In an earlier study [29], the *Synechocystis* sp. PCC 6701 *cpcA* and *cpcB* genes were expressed in a *Synechocystis* sp. PCC 6803 strain, 4R, which lacks phycocyanin due to an amber mutation in the *cpcB* gene. While the *cpcA* gene is intact in this strain, no detectable CpcA accumulates [30]. A site-directed mutation converting ^85^C to ^85^A in the heterologously expressed *Synechocystis* sp. PCC 6701 CpcA protein yielded the CD3 mutant. Since the CD3 strain lacks the phycocyanobilin binding site on CpcA this mutant is functionally equivalent to the Δ*cpcF* strain. The CD3 mutant accumulated only a small amount of total phycocyanin in its phycobilisomes (about 10% of control) and 90% of the CpcA which was present in these phycobilisomes was derived from *Synechocystis* 6803 holoCpcA. Consequently, the phycobilisomes isolated from the CD3 strain appear to incorporate about 1% of *Synechocystis* 6701 apo-CpcA.

The phycobilisomes which accumulate in the Δ*cpcF* mutant appear to function poorly in the transfer of excitation energy to the allophycocyanin core and subsequently to PS II (Fig. 2 and 3). However, they do apparently functionally associate with FNR_L_. The failure to detect FNR_L_ in Band II from the CK mutant ([9]; Figs. 5C and D) suggested that only the phycocyanin proteins, and not allophycocyanin, had a high affinity to FNR_L_. One previous study has shown that FNR was found in sucrose density gradients in association with the allophycocyanin core from a phycocyanin deletion strain [31]. These authors proposed that FNR was associated with the core-proximal allophycocyanin hexamer. We suggest that, due to the smaller size of the allophycocyanin core, it migrates to a location near the top of the sucrose density gradient which is heavily contaminated by free FNR_L_. This is clearly seen in Fig. 5E. While the top of the gradient contains a large amount of FNR_L_, only a very small amount actually comigrates with allophycocyanin.

Using cryoelectron microscopy and single particle analysis, Arteni et al. [32] proposed a model suggesting that the location of FNR_L_ is located at the interface between the phycocyanin rods and the allophycocyanin core. Our data from the Δ*cpcF* mutant (Figs. 4 and 5) support this model. Using BN-PAGE, we also identified a subcomplex containing FNR_L_-CpcA-CpcB from both the WT and Δ*cpcF* strains, but not from the CK mutant. This indicates that the association of FNR_L_ and phycocyanin can be maintained even in the absence of a high concentration of phosphate, a condition in which the phycobilisome largely dissociates (Fig. 4B). Additionally this indicates that the allophycocyanin core may not be required for the interaction of FNR_L_ with the phycobilisome. The observations of decreased FNR_L_ accumulation and its degradation in the CK mutant ([9]; Figs. 4A, and 5D and E) indicates that the FNR_L_ protein is not stable in the absence of assembled phycocyanin. It should be noted, however, that the functionality of the apparent degradation products of FNR_L_, which are significantly larger than FNR_S_, has not been evaluated.

FNR has been co-purified from several thylakoid membrane protein complexes, such as cytochrome *b_6_f* complex and NDH complex, and it has been suggested that it serves as an electron donor during cyclic electron flow [33], [34]. In cyanobacterial PetH (FNR) the N-terminal domain shares high homology with CpcD, which apparently allows a significant amount of FNR_L_ to be bound to purified phycobilisomes ([35]; Figs. 5C–E). Since the artificial fusion of the N-terminal domain sequence of *petH* with the green fluorescence protein gene led to tight association of the fusion protein with phycobilisomes [36], it appears that the N-terminal domain functions to localize FNR to the phycobilisome. However, the physiological significance of this close connection between the enzyme catalyzing the final photosynthetic linear electron transport and light-harvesting complex in cyanobacteria remains unclear. A mutant which lacked FNR_L_ exhibited a higher NADP^+^/NADPH ratio [12], which supported the earlier suggestion FNR_L_ functions as a ferredoxin-NADP^+^ oxidoreductase during linear chain electron transport, supporting autotrophic growth, while FNR_S_ is a better NADPH oxidase, accumulating when linear electron transport is compromised [9]. Consequently, the association of FNR_L_ with phycocyanin is beneficial for linear electron transport. It was proposed earlier that formation of the phycobilisome-PS I trimer supercomplex was mediated by interactions with FNR [37]. In this model, FNR was placed at the core-distal phycocyanin rods. Later study indicated that binding of FNR to the phycobilisome did not affect energy distribution between PSII and PSI during state transitions [36]. Others have suggested that the association of FNR to the phycocyanin rod elements would position the enzyme in close proximity to the surface of the thylakoid membrane, facilitating its interaction with reduced ferredoxin as it is produced at the stromal surface of PS I [36]. It was also reported that the membrane localization of FNR was required for FNR-dependent cyclic electron flow induced by salt stress [38]. Our study indicates that apo-CpcA assembles into phycobilisomes in the Δ*cpcF* mutant. Although the phycocyanin rods are inefficient in transferring excitation energy to the allophycocyanin core (Figs. 2–3), the apo-CpcA-containing phycobilisomes appear to associate with FNR, stabilizing FNR_L_ (Figs. 4–5). This would yield a direct benefit to the Δ*cpcF* strain when grown under autotrophic growth conditions (Fig. 1).

CO_2_ fixation is the major electron sink for photosynthetic electron transport, as NADPH is required to produce glyceraldehyde-3-phosphate and, consequently, to regenerate RuBP. While both the Δ*cpcF* and CK mutants grew poorly autotrophically under ambient CO_2_ conditions, the inability of the CK strain to grow in an elevated CO_2_ environment, where WT and the Δ*cpcF* grow robustly (Fig. 1), is puzzling and we do not fully understand this observation. We speculate that under this growth condition, where maximum rates of CO_2_ fixation would be expected to occur, limitations in the amount of available NADPH in the CK strain due to instability of FNR_L_ could lead to a failure in the regeneration of the high amounts of ribulose 1,5-bis-phosphate required to support the high rate of CO_2_ fixation. Additionally, inadequate glyceraldehyde-3-phospate production could have an overall detrimental effect on photoautotrophy.

## Conclusions

In the absence of a functional α-phycocyanin subunit lyase in the Δ*cpcF* mutant apo-CpcA accumulates and is integrated into modified phycobilisomes. While these phycobilisomes were unable to efficiently transfer excitation energy to PS II, they were able to bind and stabilize FNR_L_. These studies highlight the important role played by phycocyanin both in energy transfer and in the modulation of efficient linear electron transport via FNR.

## Supporting Information

Figure S1 **Quantification standards for the CpcA, CpcB, ApcB and FNR Proteins.** The indicated amounts of wild-type protein samples were resolved by LiDS-PAGE, electroblotted onto PVDF membranes, blocked and probed with antibodies against CpcA, CpcB, ApcB or FNR. After incubation with an anti-rabbit IgG-horseradish peroxidase conjugate, the blots were developed using chemilumenescence and detected by exposure to X-ray film. The X-ray film was scanned and the protein amounts were semi-quantified by comparison of the integrated optical density with a dilution series of wild-type samples (5–75 µg protein). Signal intensities were analyzed by ImageJ [19]. The R^2^ values for the linear regression of each standard curve are shown. Both FNR_L_ and FNR_S_ were detected with the anti-FNR antibody.
(TIF)

Figure S2 **Chlorophyll fluorescence induction of WT and Δ** ***cpcF*** **under low light illumination.** The cells were grown autotrophically at ambient CO_2_. Chlorophyll fluorescence induction of dark-adapted cells at a chlorophyll concentration of 5 µg/ml in BG-11 medium was measured using orange-red light (625 nm) at either 1000 µmol photons/m^2^·s (100%) or 500 µmol photons/m^2^·s (50%). The chlorophyll fluorescence was presented either as (A) the raw fluorescence traces or (B) normalized using the equation Fluorescence  =  (1-F_0_/F(t))/(F_V_/F_M_). The samples for the measurements contained no additions. The error bars represent ±1.0 SD and in some instances were smaller than the symbols; each experiment is the average of 3 independent measurements.
(TIF)
