# Supplementary figures and images for: Integration of Apo-α-Phycocyanin into Phycobilisomes and Its Association with FNRL in the Absence of the Phycocyanin α-Subunit Lyase (CpcF) in Synechocystis sp. PCC 6803
Source: PLoS One. 2014 Aug 25;9(8):e105952. doi: 10.1371/journal.pone.0105952 (PMC4143364; doi:10.1371/journal.pone.0105952)

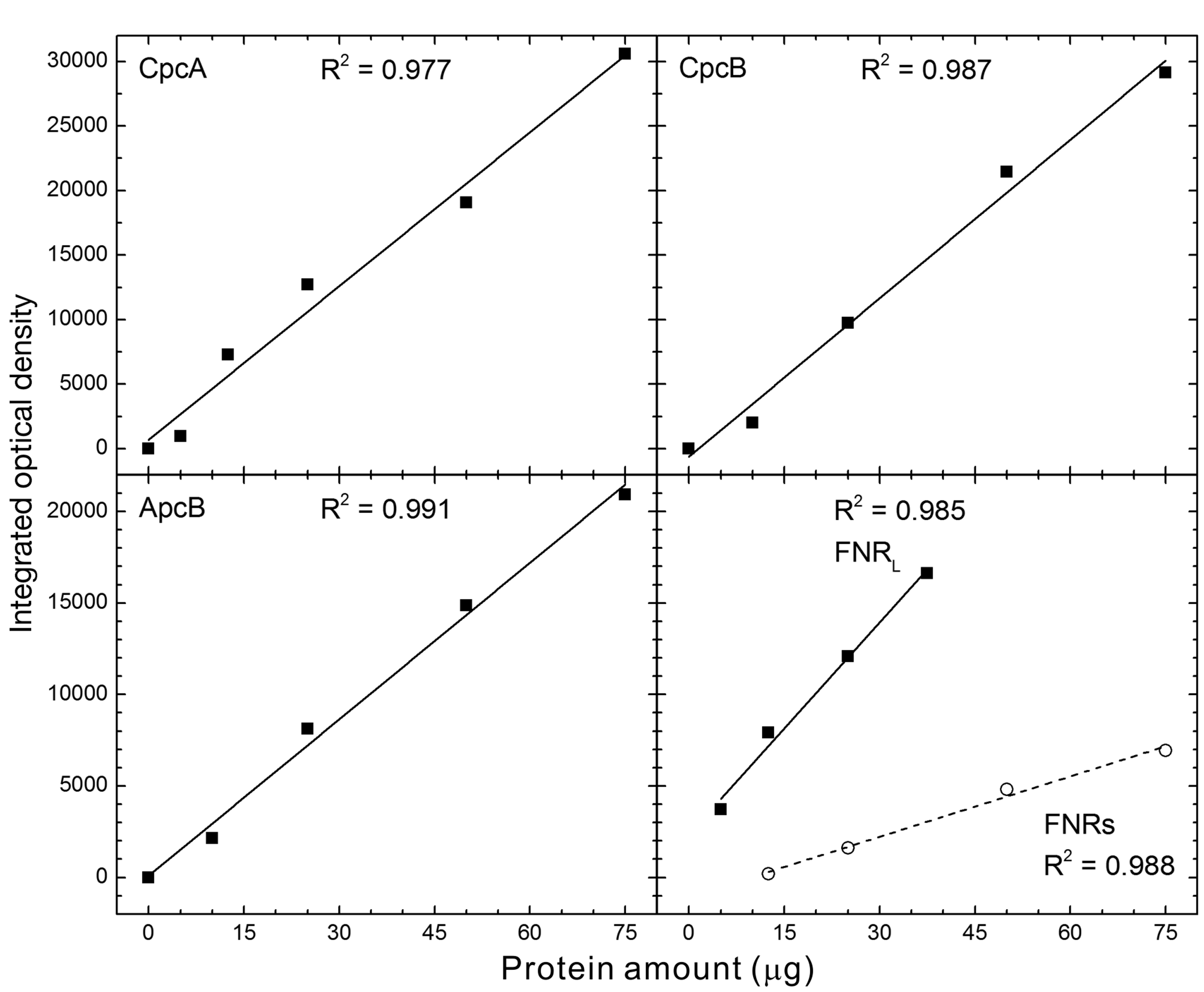

Supplement: Figure S1 — Quantification standards for the CpcA, CpcB, ApcB and FNR Proteins. The indicated amounts of wild-type protein samples were resolved by LiDS-PAGE, electroblotted onto PVDF membranes, blocked and probed with antibodies against CpcA, CpcB, ApcB or FNR. After incubation with an anti-rabbit IgG-horseradish peroxidase conjugate, the blots were developed using chemilumenescence and detected by exposure to X-ray film. The X-ray film was scanned and the protein amounts were semi-quantified by comparison of the integrated optical density with a dilution series of wild-type samples (5–75 µg protein). Signal intensities were analyzed by ImageJ [19]. The R2 values for the linear regression of each standard curve are shown. Both FNRL and FNRS were detected with the anti-FNR antibody. (TIF) [file pone.0105952.s001.tif]

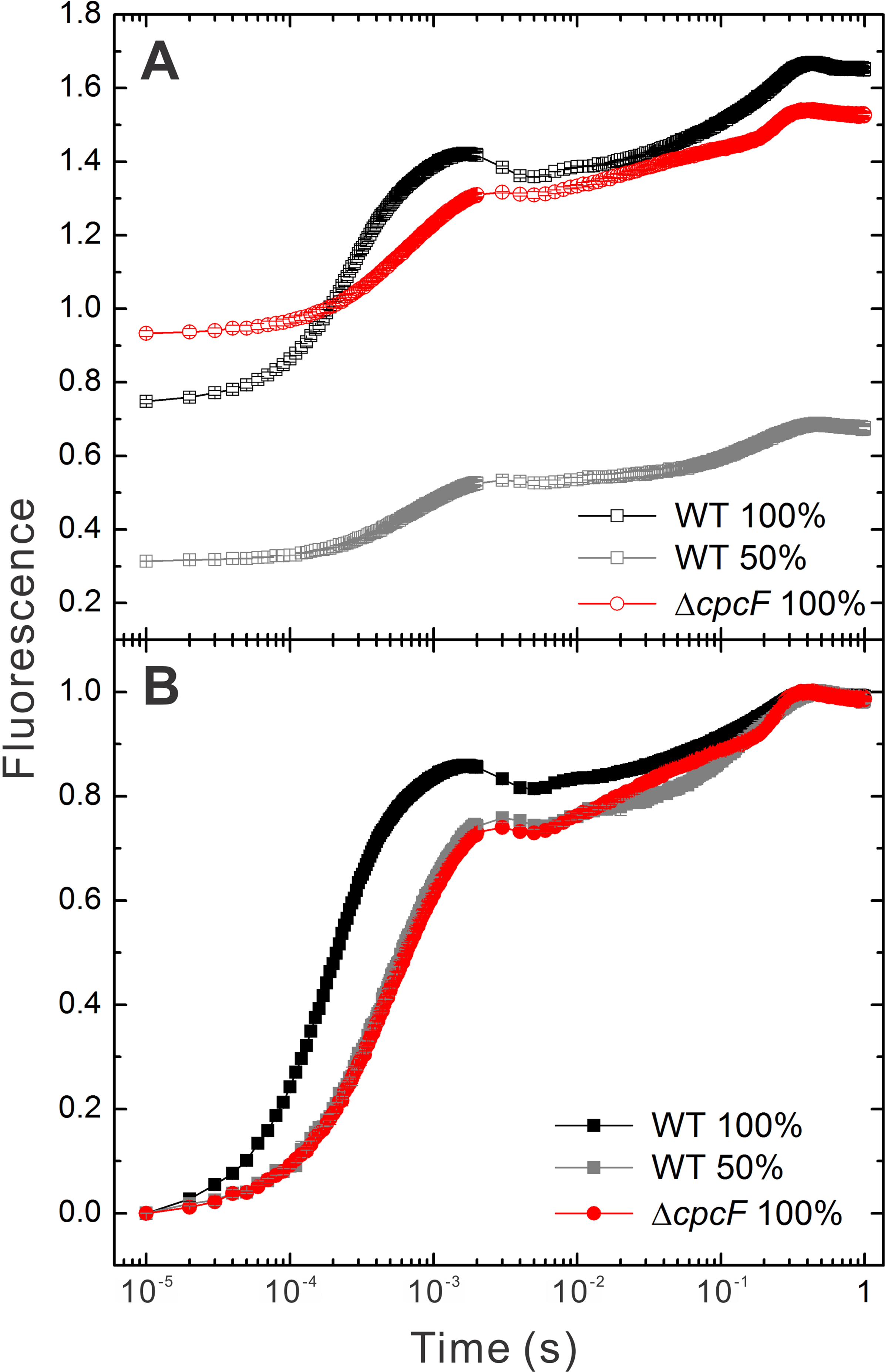

Supplement: Figure S2 — Chlorophyll fluorescence induction of WT and Δ cpcF under low light illumination. The cells were grown autotrophically at ambient CO2. Chlorophyll fluorescence induction of dark-adapted cells at a chlorophyll concentration of 5 µg/ml in BG-11 medium was measured using orange-red light (625 nm) at either 1000 µmol photons/m2·s (100%) or 500 µmol photons/m2·s (50%). The chlorophyll fluorescence was presented either as (A) the raw fluorescence traces or (B) normalized using the equation Fluorescence = (1-F0/F(t))/(FV/FM). The samples for the measurements contained no additions. The error bars represent ±1.0 SD and in some instances were smaller than the symbols; each experiment is the average of 3 independent measurements. (TIF) [file pone.0105952.s002.tif]
